# No Association between Depression and Risk of Hepatocellular Carcinoma in Older People in Taiwan

**DOI:** 10.1155/2013/901987

**Published:** 2013-12-10

**Authors:** Shih-Wei Lai, Cheng-Li Lin, Kuan-Fu Liao, Wen-Chi Chen

**Affiliations:** ^1^School of Medicine, China Medical University, Taichung 404, Taiwan; ^2^Department of Family Medicine, China Medical University Hospital, Taichung 404, Taiwan; ^3^Department of Public Health, China Medical University, Taichung 404, Taiwan; ^4^Management Office for Health Data, China Medical University Hospital, Taichung 404, Taiwan; ^5^Graduate Institute of Integrated Medicine, China Medical University, Taichung 404, Taiwan; ^6^Department of Internal Medicine, Taichung Tzu Chi General Hospital, No. 66, Sec. 1, Fongsing Road, Tanzi District, Taichung 427, Taiwan; ^7^Department of Urology, China Medical University Hospital, Taichung 404, Taiwan

## Abstract

*Objectives*. The objective of this study was to determine whether there is a relationship between depression and risk of hepatocellular carcinoma (HCC) in older people in Taiwan. *Methods*. A case-control study was conducted to analyze the database from the Taiwan National Health Insurance program. We selected 1815 subjects aged 65 years or older with newly diagnosed HCC as the case group and 7260 subjects without HCC as the comparison group, from 2000 to 2010. Both groups were compared to measure the risk of HCC. *Results*. After controlling for confounders, the odds ratio of HCC was 0.81 in subjects with depression (95% confidence interval = 0.59, 1.11), as compared with nondepressed subjects. *Conclusions.* We conclude that no association is detected between depression and risk of hepatocellular carcinoma in older people in Taiwan.

## 1. Introduction

Until now, controversies exist regarding the relationship between depression and subsequent cancer risk. That is, some studies showed an increased risk [1, 2], but others showed no increased risk [3, 4]. Similarly, no specific evidence is available about the relationship between depression and risk of hepatocellular carcinoma (HCC) in older people. In order to explore this issue, a population-based case-control study was conducted to analyze the database from the Taiwan National Health Insurance program.

## 2. Methods

The details of the Taiwan National Health Insurance program can be documented in previous studies [5–7]. In this case-control study, we randomly selected 1815 subjects aged 65 years or older with newly diagnosed HCC as the case group (1132 men and 683 women, mean age 74.31 years, and standard deviation 6.29 years) (according to the International Classification of Diseases 9th Revision-Clinical Modification, ICD-9 codes 155, 155.0, and 155.2) and 7260 subjects without HCC as the comparison group (4528 men and 2732 women, mean age 74.09 years, and standard deviation 6.53 years). Both groups were matched with sex, age, and index year of diagnosing HCC, from 2000 to 2010. The index date was defined as the date of diagnosing HCC. In order to reduce the confounding effects, subjects with any cancer (ICD-9 codes 140–208), major psychiatric diseases (ICD-9 codes 291–293, 294.0, 294.8-294.9, 295, 296.0-296.1, 296.4–296.9, and 297-298), other dementia (290.0–290.4, and 294.1), or mental retardation (ICD-9 codes 317–319) diagnosed before the index date were excluded. Depression (ICD-9 codes 331.0) and other comorbidities potentially associated with HCC were diagnosed before the index date [7].

## 3. Results

We compared the basic characteristics and co-morbidities between the HCC group and the comparison group.  Table 1 shows that the HCC group was more likely to have diabetes mellitus, cirrhosis, other chronic hepatitis, hepatitis B infection, hepatitis C infection, and alcoholism, with statistical significance. There were 114 subjects with depression among the HCC group (6.28%) and 385 subjects with depression among the comparison group (5.30%), without significant difference (*P* = 0.10). After controlling for confounding factors, multivariable logistic regression analysis demonstrated that the odds ratio (OR) of HCC was 0.81 in subjects with depression (95% confidence interval (CI) = 0.59, 1.11), as compared with nondepressed subjects (Table 2).

## 4. Discussion

To date, there is no consensus about the association between depression and subsequent cancer risk [1–4]. One cohort study by Chen and Lin in Taiwan showed that patients with depression correlate with 1.8-fold increased risk of gastrointestinal cancer (95% CI = 1.03, 3.14), but HCC is not mentioned specifically [2]. In the present study, despite the fact that we controlled the comorbidities potentially associated with HCC, no association is detected between depression and HCC risk in older people (OR = 0.81, 95% CI = 0.59, 1.11). Generally speaking, 70–90% of all HCC cases are significantly associated with cirrhosis or other chronic the liver diseases worldwide [8]. Depression is a mood disorder, not causing chronic inflammation of liver. Therefore, these two diseases should have their unique pathogenesis without potential linking. Since formal depression diagnostic procedures were not recorded in this database, more prospective studies are necessary to validate our findings.

## Figures and Tables

**Table 1:** Characteristics between hepatocellular carcinoma group and comparison group.

	Hepatocellular carcinoma				*P* value
	No		Yes		
	*N* = 7260		*N* = 1815		
	*n*	(%)	*n*	(%)	
Age group (year)					
65–74	4260	58.68	1065	58.68	0.99
75–84	3000	41.32	750	41.32	
Sex					
Men	4528	62.37	1132	62.37	0.99
Women	2732	37.63	683	37.63	
Comorbidities before index date*					
Depression	385	5.30	114	6.28	0.10
Diabetes mellitus	1941	26.74	636	35.04	<0.0001
Cirrhosis	137	1.89	990	54.56	<0.0001
Other chronic hepatitis	1163	16.02	1033	56.91	<0.0001
Hepatitis B infection	112	1.54	361	19.89	<0.0001
Hepatitis C infection	135	1.86	600	33.06	<0.0001
Alcoholism	57	0.79	24	1.32	0.03

Data are presented as the number of subjects in each group, with percentages given in parentheses.

Chi-square test comparing subjects with and without hepatocellular carcinoma.

*Comorbidities included before index date were as follows: depression (ICD-9 codes 296.2-296.3, 300.4, and 311), diabetes mellitus (ICD-9 codes 250), cirrhosis (ICD-9 codes 571.2, and 571.5-571.6), other chronic hepatitis (ICD-9 codes 571.40-571.41, 571.49, and 571.8-571.9), hepatitis B infection (ICD-9 codes V02.61, 070.20, 070.22, 070.30, and 070.32), hepatitis C infection (ICD-9 codes V02.62, 070.41, 070.44, 070.51, and 070.54), and alcoholism (ICD-9 codes 303, 305.00, 305.01, 305.02, 305.03, and V11.3).

**Table 2:** Odds ratio and 95% confidence interval of hepatocellular carcinoma associated with depression and other comorbidities.

Variable	Crude		Adjusted^†^	
	OR	(95% CI)	OR	(95% CI)
Age (per one year)	1.01	(1.00, 1.01)	1.04	(1.03, 1.05)
Comorbidities before index date (yes versus no)				
Depression	1.20	(0.97, 1.49)	0.81	(0.59, 1.11)
Diabetes mellitus	1.48	(1.33, 1.65)	1.04	(0.89, 1.22)
Cirrhosis	62.39	(51.46, 75.65)	28.55	(23.17, 35.19)
Other chronic hepatitis	6.93	(6.19, 7.75)	2.23	(1.89, 2.63)
Hepatitis B infection	15.84	(12.72, 19.73)	5.87	(4.40, 7.84)
Hepatitis C infection	26.06	(21.42, 31.72)	7.72	(6.00, 9.93)
Alcoholism	1.70	(1.05, 2.74)	0.85	(0.43, 1.68)

^†^Adjusted for age, diabetes mellitus, cirrhosis, other chronic hepatitis, hepatitis B infection, hepatitis C infection, and alcoholism.
